# Preliminary evaluation of an extract of *Amaranthus caudatus* as a novel nutraceutical: effects on cholesterol and glucose absorption

**DOI:** 10.3389/fnut.2025.1740097

**Published:** 2026-01-12

**Authors:** Alessia Mattiello, Gloria Barbaglio, Mirko Magnone, Sonia Scarfì, Stefania Vernazza, Serena Mirata, Jan Markus, Silvia Letasiova, Danilo Riva, Roberto Premoli

**Affiliations:** 1 NatIng Italia Srl, Casalpusterlengo, Lodi, Italy; 2 DIMES, University of Genova, Genova, Italy; 3 DISTAV, University of Genova, Genova, Italy; 4 Inter-University Centre for the Promotion of the 3Rs Principles in Teaching & Research, Pisa, Italy; 5 MatTek In Vitro Life Science Laboratories, Bratislava, Slovakia; 6 Nova Argentia, Gorgonzola, Milan, Italy

**Keywords:** *Amaranthus caudatus*, antioxidant activity, cholesterol absorption, glucose absorption, glycemic control, metabolic syndrome, nutraceutical

## Abstract

**Introduction:**

The increasing prevalence of metabolic syndrome has prompted the development of nutraceuticals to support the management of glucose intolerance and dyslipidemia. *Amaranthus caudatus* is a potential source of bioactive compounds with metabolic relevance.

**Methods:**

A novel ingredient derived from *Amaranthus caudatus* seeds was characterized by untargeted metabolomic analysis and bioinformatic processing. Its effects were evaluated through *in vitro* enzyme inhibition and intestinal absorption assays, as well as preliminary observations in subjects with glucose intolerance over 3 months.

**Results:**

The extract significantly inhibited key enzymes involved in carbohydrate metabolism and reduced glucose and cholesterol absorption in a three-dimensional intestinal model, while showing antioxidant activity. Preliminary human observations indicated improvements in HbA1c, post-prandial glucose, BMI, and a trend toward reduced total and LDL cholesterol.

**Discussion:**

These results suggest that *Amaranthus caudatus* seed extract may represent a promising nutraceutical for supporting glycemic control and lipid metabolism.

## Graphical abstract

**Figure:**
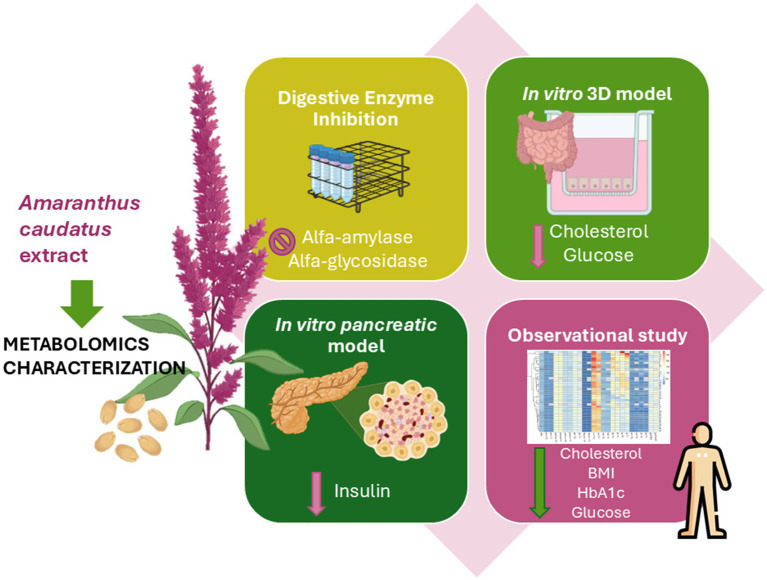

## 1 Introduction

In recent years, interest in plant-based raw materials for food and medical application has grown significantly (1). Various cereal grains are widely used in the food and beverage industry. Among these plants, a notable group classified as pseudo-cereals produces edible seeds that are consumed similarly to traditional cereals and are often processed into flour. Different regions favor different pseudo-cereals, and today, they remain a crucial part of the diet in the world’s poorest areas while gaining popularity in European countries. The best-known pseudo-cereals are buckwheat, sorghum, millet, chia, and khorasan and amaranth.

Amaranth (Amaranthus spp.) is a dicotyledonous species, member of the Amaranthaceae family, native of the Andean regions and cultivated in different countries in South and Central America, Africa, India, and Asia (2–4). It is renowned for its excellent nutrient profile as well as its rich phytochemical composition. Amaranth is a fast-growing crop with low production cost, making it one of the cheapest dark green vegetables in the tropical market, often referred to as the poor man’s vegetable. Unlike other green vegetables, it is cultivated during summer when no other green vegetables are available in the market (5). Such pseudo-cereal can be used to produce gluten-free cereal-based products, and it is considered a good candidate for supplementing or substituting common cereal grains such as rye, oat or wheat (6, 7).

It is characterized by a high content of high-quality protein and essential amino acids such as lysine, cysteine and methionine and it consists of an important source of micronutrients (minerals, vitamins, flavonoids, phytosterols, and polyphenols) (6, 8). In particular, the protein content of amaranth is about 18%, higher than the one of traditional cereal, though this can vary based on environmental factors like climate, soil conditions and others (1, 9). As for carbohydrates, starch is the main one with a percentage of 45–65%. Lipids (~7%) is the principal source of nutritional value and they are represented mainly by linoleic, oleic and linolenic acid, meanwhile the saturated fatty acids are present in small amounts.

Thanks to these features, amaranth-based products consumption is associated with various nutritional benefits and health advantages (10). As a source of lipophilic antioxidants, particularly squalene and phenolic compounds like ferulic acid, amaranth has marked antioxidant activity: extracts of amaranth seeds have demonstrated strong antioxidant effects in cell models, particularly against the superoxide radical (11). Additionally, amaranth extracts show anti-inflammatory properties; for instance, selenium and betacyanins in edible amaranth seed sprouts have been shown anti-inflammatory effect by significantly decrease the pro-inflammatory cytokine IL-6, as demonstrated by Tyszka-Czochara et al. (12). Moreover, Gullón et al. demonstrated how the prebiotic potential of amaranth could be added as a premise for improving or maintaining gastrointestinal health through the equilibrium of intestinal microbiota (2). Other properties related to amaranth, and in line with this study, are those related to anti-obesity and anti-diabetic activities. Amaranth seeds could, indeed, function as a protective agent against fructose-induced obesity and diabetes mellitus (13). Amaranth seeds are also beneficial for correcting complications related to Metabolic Syndrome such as hyperglycemia and diabetic complications (10).

Metabolic syndrome comprises a series of cardiometabolic risk factors, including central obesity, high blood sugar levels, high blood pressure, and abnormal cholesterol levels (14). The co-occurrence of these factors significantly increases the risk of developing type 2 diabetes (T2D) and cardiovascular diseases.

T2D is a progressive disease, starting from impaired glucose tolerance, with decline in *β*-cell function and worsening of insulin resistance, deteriorations in glycated hemoglobin (HbA1c), fasting plasma glucose and postprandial glucose levels (15). The progression typically moves from normal glucose tolerance to impaired fasting glucose (IFG) and/or impaired glucose tolerance (IGT), eventually leading to the diabetic condition (16). In the general population, a lifestyle characterized by increased caloric intake and reduction of physical activity led to a rise in the prevalence of overweight and obesity conditions. Excess weight exerts a crucial effect on glucose tolerance, increasing the risk of progressing to T2D. This risk escalates progressively with increasing Body Mass Index (BMI), even within the range of BMI values considered normal. High BMI values are also associated with elevating blood levels of total cholesterol and LDL cholesterol, substantially increasing the risk for cardiovascular disease (17).

Considering its use as a commercial functional ingredient, AmaChol^®^—an extract derived from *Amaranthus caudatus*—is widely promoted for metabolic health. However, despite its market availability, the scientific evidence supporting its purported metabolic effects remains incomplete. The current literature provides fragmented information on its bioactive constituents, with insufficient mechanistic characterization and no integrated assessment across experimental models. This gap limits any rigorous evaluation of its efficacy and hinders a clear understanding of the biological pathways potentially involved. Therefore, the aim of this study was to determine, through a preliminary experimental investigation, the metabolomic composition of the specific extract, whether it exerts measurable effects on glucose and lipid metabolism and to elucidate the mechanistic basis of these actions using complementary *in vitro* and *in vivo* approaches. For this purpose, a detailed analysis of the metabolites present in the extract was performed using LC–MS/MS approach, accompanied by rigorous in vitro investigations and preliminary studies in patients, in order to determine whether the extract influences key processes relevant to metabolic syndrome, including glucose handling, cholesterol absorption, and oxidative balance.

## 2 Materials and methods

### 2.1 Materials

Alpha-amylase, pepsin, bile extract, pancreatin, maltodextrin, Glucose (GO) assay kit were purchased from Merck Life science (Milan, Italy); BODIPY-cholesterol (Cayman Chemical) were purchased from Cabru S.a.s. (Milan, Italy); Normal human 3D small intestinal Epithelium (Epiintestinal^®^) and maintenance medium were purchased from MaTtek (Bratislava, Slovakia); human pancreatic islets and appropriate media for their culture were purchased from Tebu-Bio S.r.l. (Milan, Italy); ELISA kit for insulin assay (Mercodia) was purchased from D.B.A. Italia (Milan, Italy); Falcon^®^ sterile plasticware was purchased from Fisher Scientific (Milan, Italy); ADMEM/F12, Phosphate saline buffer (PBS), Hanks’ Balanced Salt Solution (HBSS) were purchased from Euroclone (Milan, Italy).

### 2.2 *Amaranthus caudatus* extract preparation

The *Amaranthus caudatus* L. dry extract (AmaChol^®^) is a semi-finished botanical product supplied by Nating Italia S.r.l. (Batch: NAT20251736) and intended for the formulation of food supplements. The extract is obtained from *Amaranthus caudatus* seeds through a straightforward aqueous extraction process. Briefly, seeds are thoroughly cleaned to remove impurities and coarsely ground to increase surface area and facilitate extraction. The ground seed material is combined with distilled water, typically in a 1:10 solid-to-liquid ratio. This mixture is gently heated to a temperature range of 70–80 °C. The heating step is maintained for 30 to 60 min with occasional stirring to enhance extraction efficiency. After heating, the mixture is allowed to cool at room temperature. Once cooled, it is filtered to separate the solid residues from the liquid phase and subsequently spray-dried to obtain a powdered dry extract. The dry extract was stored at 25 °C under controlled humidity in an ISO 9001:2015-certified facility ensuring standardized workflows, traceability, and quality control in accordance with standard industrial storage conditions for semi-processed herbal materials.

### 2.3 Characterization of Amaranthus extract through untargeted metabolomics

The metabolic profiles of the powdered dry extract were analyzed using an Ultimate 3,000 UPLC system (Thermo Fisher Scientific) coupled with a high-resolution Q-Exactive Plus Hybrid Quadrupole-Orbitrap™ mass spectrometer (Thermo Fisher Scientific). Sample preparation involved ultrasonication of 500 mg of semi-finished dry extract in 3 mL of chloroform/methanol (2:1) for 20 min, followed by centrifugation at 4,000 rpm for 15 min. The aqueous phase was separated from the organic phase. The first one was directly injected into the UPLC-Q-Exactive Plus system without any dilution. Differently, the organic phase was dried under a nitrogen stream and resuspended in 1 mL of a solution including acetonitrile/isopropanol/methanol (2/5/3). Mass spectrometry data were acquired in both positive and negative ionization modes over an m/z range of 200–2000. High-resolution mass spectra were obtained using the following parameters: spray voltage of 3.5 kV (positive) and 3.0 kV (negative), sheath gas at 20 arbitrary units, auxiliary gas at 5 arbitrary units, capillary temperature at 320 °C, and a resolution of 35,000. The acquisition mode was set to Full MS/dd-MS^2^-Top N, where precursor ion selection and fragmentation were based on ion abundance. MS/MS spectra were generated using Higher-energy Collisional Dissociation (HCD) with a collision energy of 30 arbitrary units, and a mass accuracy threshold of 5 ppm was applied. Chromatographic separation was achieved using an Acquity UPLC BEH C18 column (2.1 mm × 150 mm, 1.7 μm, Waters). The mobile phase consisted of solvent A (0.1% formic acid in water) and solvent B (0.1% formic acid in acetonitrile), both from Merck Group. The gradient elution started with 2% B, held constant for 1 min, then gradually increased to 100% B over 50 min, maintained for 2 min, and finally returned to initial conditions. The flow rate was set at 0.2 mL/min, the injection volume was 10 μL, and the column temperature was maintained at 35 °C. The analysis was performed in technical triplicate. No biological replicates were included, as the study focused on metabolomic characterization of semi-finished botanical extract. Data normalization and scaling procedures were not applied, as the study was designed to provide a qualitative and semi-quantitative metabolomic characterization of a single semi-finished botanical product rather than comparative or inferential analyses. Peak areas were therefore used solely to support metabolite detection and annotation, and not for quantitative comparison across samples. No pooled quality control (QC) samples were included; consequently, the interpretation of the metabolomic data is limited to compound annotation and relative abundance assessment. Raw LC–MS/MS data were processed using Compound Discoverer 3.3 (Thermo Fisher Scientific). Feature detection and alignment were performed with default settings, except for a retention-time tolerance of 0.2 min and a mass tolerance of 10 ppm. Blank solvent runs were acquired under identical conditions to identify and exclude background peaks originating from the matrix or solvent, thereby enhancing annotation reliability. The workflow included automated feature detection, chromatographic alignment, background subtraction, isotope/adduct grouping, and compound annotation. For each detected feature, CD returned (when available) the compound name, molecular formula, precursor m/z, calculated molecular weight, retention time (RT), maximum peak area, ionization mode (ESI positive or negative), and MS/MS-based annotation obtained through matching against the spectral and structural databases integrated into the platform (mzCloud, mzVault, ChemSpider, Mass List, Metabolika). The full feature table exported from CD containing these parameters for all detected compounds are provided as Supplementary materials. In line with the Metabolomics Standard Initiative (MSI), features annotated on the basis of accurate mass and MS/MS spectral similarity to database entries are classified as MSI Level 2. The MSI confidence level associated with each feature is explicitly indicated in the tables provided in the Supplementary materials. As such, non–standard-confirmed metabolites discussed in the main text should be regarded as putative annotations (MSI Level 2) pending further validation with authentic standards.

- Database Confirmation: Compounds showing full or partial correspondence in at least one of five reference databases (m/z Cloud, m/z Vault, Metabolika, ChemSpider, or Mass List) were included.
- Mass Accuracy: Deviation within ±3 ppm from theoretical m/z.
- RT (Retention Time): Compounds eluting between 5 and 50 min were selected, although the range could be extended (0–120 min) to accommodate specific analytical requirements.
- Peak Area: Features with areas below 1.0 × 10–5 were excluded in order to minimize low-intensity background signals.
- MS2 Availability: Only compounds with corresponding MS2 spectra were retained for annotation.

The resulting dataset was cross-checked against the published literature to verify compound identities and contextualize the detected metabolites within known phytochemical profiles. For structural classification of annotated metabolites, Graph Isomorphism Network (GIN) models were employed as a complementary in silico tool to support compound class assignment. GIN-based classification was performed according to the framework described in Prete et al. (18). GINs were selected due to their ability to capture molecular graph topology with high fidelity and to support chemical class assignment in natural product datasets. Each metabolite was converted from its SMILES notation into a molecular graph representation, where atoms were encoded as nodes with associated features (atom type, degree, hybridization state, formal charge, aromaticity) and bonds were encoded as edges with features describing bond type and conjugation. The GIN output was used exclusively to support the assignment of broad structural classes and to facilitate chemical organization of the metabolite dataset. No *de novo* structure prediction, quantitative inference, or biological interpretation was derived from the GIN-based classification, which was considered complementary to MS/MS-based annotation.

### 2.4 Enzymatic assays

#### 2.4.1 Assessment of the inhibition of *α*-amylase

α-amylases catalyze the hydrolysis of 1,4 *α*-D-glucosidic linkages present in polysaccharides such as starch or glycogen. α-amylase activity is measured by a colorimetric assay using 3,5-dinitrosalicylic acid (DNS) based on the following reaction, as described in the Equation 1,


(1) $$\text{Starch}\;(\begin{array}{l} \alpha -1,\\ 4-\text{glucan} \end{array})+n\cdot H_{2}O\overset{\alpha -\text{amylase}}{\to }\text{Reducing}\;\text{sugars}\;(\begin{array}{l} \text{mainly}\;\\ \text{maltose} \end{array})$$

The released maltose reduces 3,5-dinitrosalicylic acid resulting in a yellow to orange/red color change. The intensity of the color change is proportional to the amount of reducing sugars released and is measured spectrophotometrically at 540 nm. α-amylase activity is defined as follows: one unit of enzyme produces 1 mg of maltose from starch in 3 min at pH 6.9 and 20 °C. The results for the tested product were compared with the enzyme activity per se. A positive inhibition control (acarbose at 1 mg/mL) was used as a positive control for enzymatic inhibition of carbohydrate hydrolysis.

#### 2.4.2 Assessment of the inhibition of *α*-glycosidase

α-glycosidase is a hydrolase-class enzyme that catalyzes the hydrolysis of maltose, a disaccharide composed of two glucose molecules. It catalyzes the hydrolysis of the terminal, non-reducing, 1–4 linked residue of *α*-D-glucose with the release of α-D-glucose. The α-glycosidase activity is measured by a colorimetric assay using p-(4-nitrophenyl)-α-D- glucoside, as described in the Equation 2.

The assay is based on the following reaction:


(2) $$\begin{array}{l} p-(\begin{array}{l} 4-\\ \text{Nitrophenyl} \end{array})-\alpha -D-\text{glucoside}\overset{\alpha -\text{glycosidase}}{\to }\alpha -D-\text{glucose}+\\ p-\text{Nitrophenol} \end{array}$$

The released nitrophenol causes a color change towards yellow. The intensity of the color change is proportional to the activity of the enzyme and is measured spectrophotometrically at 400 nm. *α*-glycosidase activity is defined as follows: one unit of enzyme produces 1 μmol of D-glucose per minute from p-(4-nitrophenyl)-*α*-D-glucoside at pH 6.8 and 37 °C. The results for the tested (potentially inhibiting) products were compared with the enzyme activity in the absence of inhibitor. A positive inhibition control (acarbose at 1 mg/mL) was used as a positive control for enzymatic inhibition of carbohydrate hydrolysis.

### 2.5 Assessment of antioxidant activity

The antioxidant potential of the ingredient was analyzed by a colorimetric assay measuring the capacity of preventing 2,2′-Azino-bis (3-ethylbenzothiazoline-6-sulfonic acid) (ABTS) oxidation, at different concentrations. Colorimetric reactions were per- formed in a final volume of 200 μL in sodium acetate buffer, 50 mM, pH 4.5, containing: ABTS (15 mM); HRP (2.5 μM); H_2_O_2_ (0.4 mM) as an oxidizing agent.

Samples at different concentrations were added to a mixture containing sodium acetate buffer, ABTS (15 mM) and HRP (2.5 μM), in the wells of a 96-well multiwell plate, and the initial absorbance was measured at 415 nm. At the end of the first reading, the solution containing H_2_O_2_ (0.4 mM) was added to each well, and the plate was incubated at room temperature, in the absence of light, for 5 min. At the end of the incubation, the absorbance at 415 nm of each sample was read.

The difference between the final and initial absorbance is indicative of ABTS oxidation and is inversely proportional to the antioxidant power of the compound used.

### 2.6 Simulation of gastrointestinal digestion *in vitro*

Maltodextrin and *Amaranthus caudatus* extract were digested according to the experimental protocol described by Minekus et al. (19). Briefly: 300 mg of each compound was resuspended in 7.5 mL of Simulated Salivary Fluid (SSF) 1.25x, 10 mg of alpha-amylase dissolved in 0.5 mL of SSF 1.25x, 0.025 mL of 300 mM CaCl_2_ and ultrapure water obtained by milliQ system (Millipore, Germany) until 10 mL final volume was reached. The suspension was kept in agitation at 37 °C for 2 min and then fortified with 7.5 mL Simulated Gastric Fluid (SGF) 1.25x, 12.5 mg pepsin dissolved in 1.6 mL SGF 1.25x, 0.2 mL HCl 1 M, 0.005 mL CaCl_2_ 300 mM, 0.695 mL milliQ ultrapure water and left in agitation at 37 °C for 2 h. At the end, the suspension was further enriched with 11 mL Simulated Intestinal Fluid (SIF) 1.25x, 40 mg pancreatin dissolved in 5 mL SIF 1.25x, 163.5 mg bile extract resuspended in 2.5 mL SIF 1.25x, 0.04 mL CaCl_2_ 300 mM, 0.15 mL NaOH 1 M and 1.31 mL ultrapure water milliQ. The suspension was kept in agitation at 37 °C for 2 h.

At the end of incubation, each suspension containing the liberated glucose was centrifuged and the supernatant subjected to enzymatic glucose assay using the Glucose (GO) Assay kit. The method is based on the specific oxidation of glucose to gluconic acid and hydrogen peroxide catalyzed by glucose oxidase. The hydrogen peroxide produced subsequently reacts with o-dianisidine in the presence of peroxidase, yielding a colored oxidized chromogen. To stabilize the chromophore, sulfuric acid was added, converting the oxidized o-dianisidine into a stable pink-colored product. Absorbance was measured spectrophotometrically at 540 nm. The absorbance signal was directly proportional to the glucose concentration in the original sample and was used for quantitative determination. A reagent blank consisting of digestion fluids and enzymes without any carbohydrate substrate was processed in parallel to account for background glucose and reagent-derived absorbance. Maltodextrin digestion under standard enzymatic conditions was used as a positive digestion control.

### 2.7 Simulation of intestinal absorption *in vitro*

Simulation of intestinal absorption has been performed to assess the ability of the *Amaranthus caudatus* extract to reduce both glucose and cholesterol absorption. As a physiologically relevant model, normal human 3D small intestinal Epithelium (Epiintestinal^®^) was used, according to manufacturer’s protocols. Such tissues derive from postmortem small intestine cells from donors, according to the Institutional Review Board (IRB), and include enterocytes, Paneth cells, M cells, tuft cells and intestinal stem cells to reconstitute a highly differentiated and polarized epithelium (20).

#### 2.7.1 Glucose absorption

In order to assess glucose absorption, the tissues were incubated, on the apical side, with glucose or with the samples obtained by gastrointestinal digestion, or with appropriate controls. To this purpose, the tissues were subjected to preliminary starvation process for 1 h in a 37 °C incubator to decrease the endogenous glucose content (about 25 mM) present in the specific culture medium. Then, further re-equilibration of the intestinal tissues was performed for 30 min at 37 °C by placing, respectively, 200 and 500 μL of PBS with Ca^2+^ and Mg^2+^ and 1 mM glucose in the apical and basolateral sides of the Epiintestinal™ tissue. This additional rebalancing creates the correct concentration gradient suitable to promote the uptake and passage of glucose present in the samples from the apical to the basolateral region, mimicking the intestinal absorption process. Then, the incubations with the digested or undigested samples were additioned with 40 mM glucose, thus mimicking the concentration of glucose theoretically achievable in the intestinal lumen with a full meal. Untreated controls were accordingly incubated with 40 mM glucose only. The incubation was performed for 30 min in a cell culture incubator at 37 °C and 5% CO_2_. At the endpoint, the apical and basolateral solution were recovered from each 3D model tissue and the glucose present in both fractions (apical and basolateral) was quantified through the Glucose (GO) Assay kit following manufacturer’s instructions as reported above.

#### 2.7.2 Cholesterol absorption

To assess cholesterol absorption, human 3D small intestinal Epithelium was incubated, on the apical side, with *Amaranthus caudatus* extract or maltodextrin as a control for 10 min at 37 °C, and 500 μL of Hanks’ Balanced Salt Solution (HBSS) was placed in the basolateral side of each tissue. Then, a fluorescent cholesterol analogue (BODIPY-cholesterol reagent, 5 μM) was added to the apical side of each tissue, and the samples were incubated at 37° C for an additional 10 min. Following isopropanol dissolution and recovery, cholesterol absorption was measured through fluorescence spectrofluorimetric reading (Fluostar, Clario, λex 488 nm and λem 520 nm).

#### 2.7.3 Insulin release assay from human pancreatic islets stimulated with Epiintestinal^®^ basolateral fractions

Human pancreatic islets for this work (HIR^®^ Prodo Laboratories Inc., distributed by Tebu-Bio S.r.l.) were collected from organ donor pancreas and cleared for research purposes. For insulin release assay, 100 pancreatic islets per experimental point were used following the procedure briefly described below.

The pancreatic islet suspension maintained in culture flask was transferred to 15 mL sterile tube, centrifuged 180xg for 2 min, supernatant removed, and islet pellet resuspended in PBS containing 1 mM glucose. The cell suspension was then placed in a 37 °C incubator for 4 h to equilibrate the internal insulin content of the islets. After completion, further centrifugation at 180xg for 2 min was performed, the supernatant was re- moved, the pellet was resuspended in 1 mL of PBS/glucose 1 mM, and the resulting sus- pension was divided into aliquots of 100 islets/100 microliters in 10 sterile 0.5-mL tubes that were then placed in a 37 °C incubator for 30 min. Subsequently, the suspensions were allowed to settle for 30 s to deposit the islands at the bottom of each tube and re- moved the supernatant. This supernatant, recovered from each tube, was used to determine the basal insulin level. The islets were then stimulated with the basolateral fractions recovered from the Epiintestinal™ tissues obtained as described above and incubated at 37 °C for 30 min. At the endpoint, each sample was centrifuged at 180xg for 2 min and the supernatant was recovered, and insulin release was quantified through ELISA assay.

### 2.8 Preliminary observational study

A preliminary observational, real-life study was conducted for 3 months in the territory of Como Province (Italy) on sixty subjects with altered glycemic control. Participants were selected based on fasting plasma glucose values between 100 and 126 mg/dL, corresponding to impaired fasting glucose ranges according to ADA criteria; however, a formal diagnostic classification according to ADA or WHO standards was not performed, and the classification here was used for participant selection purposes only. The study aimed to evaluate the impact of the administration of an *Amaranthus caudatus* extract on the parameters of Metabolic Syndrome. They are characterized as follows:

- Gender: male and female.
- Age: between 30 and 70 years.
- Fasting plasma glucose: between 100 and 126 mg/dL.
- Body mass index (BMI): between 20.1 and 34.9 kg/m^2^.
- Medication: not subjected to hypoglycemic therapy.

**Figure 1:**
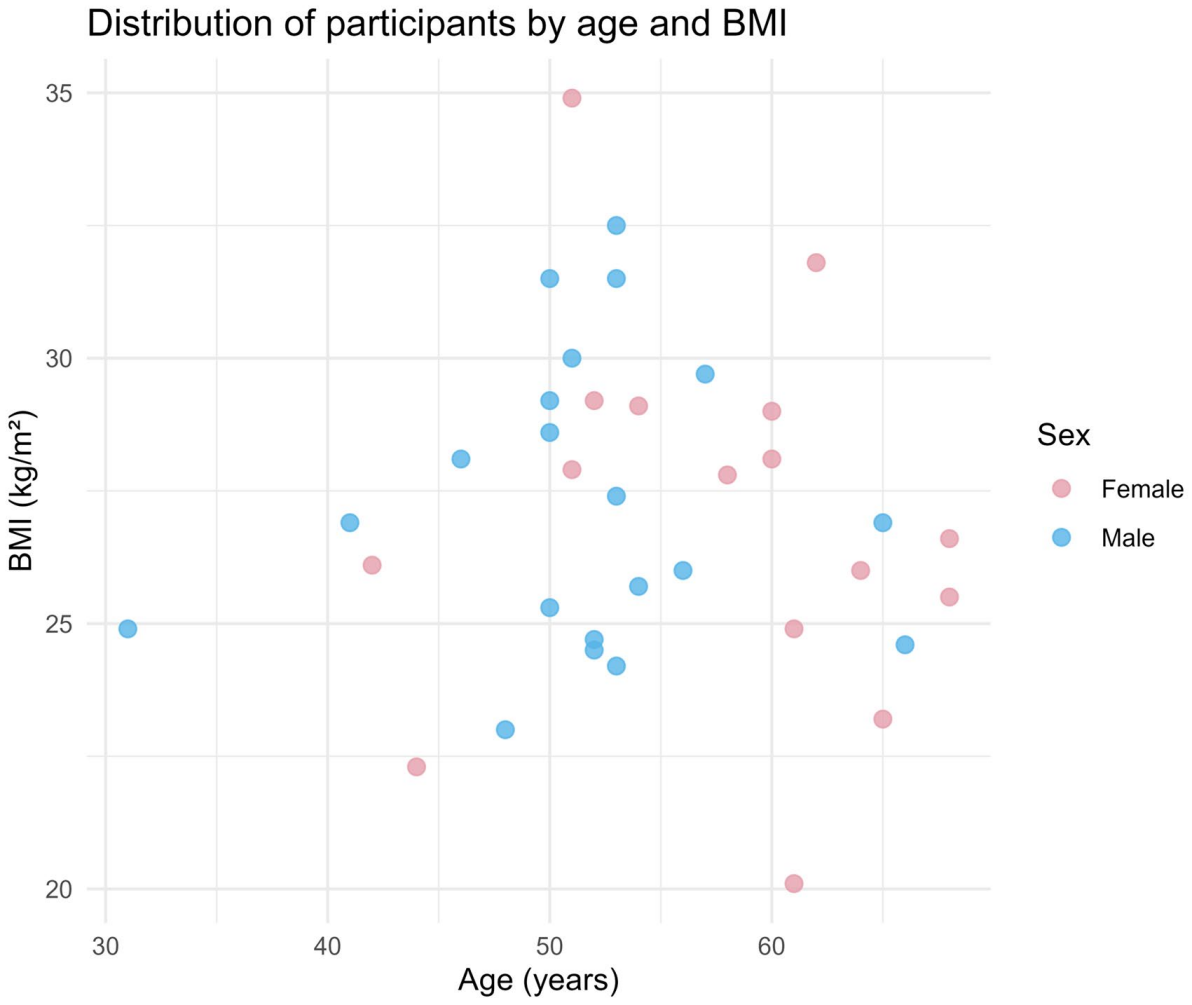
Distribution of study participants by age and body mass index (BMI). Dot plot representing individual participants, with BMI on the *y*-axis, age on the *x*-axis, and sex indicated by color. Each point corresponds to a single participant, allowing visualization of the distribution and heterogeneity of the study population.

To provide a visual overview of the heterogeneity of the study population, a dot plot was generated representing individual participants’ age (x-axis) versus BMI (y-axis), with points colored according to sex (Figure 1). Most participants were between 50 and 55 years of age and had a BMI between 26 and 30 kg/m^2^. For one female participant with a BMI of 32.2 kg/m^2^, age information was unavailable and therefore could not be represented along the x-axis in the plot. This representation allows visualization of the distribution of participants across both age and BMI, highlighting the range and overlap between male and female subjects.

The primary endpoint was reduction in glycated hemoglobin, and the secondary endpoints were reduction in postprandial blood glucose, reduction in total cholesterol, and reduction in weight and BMI (Body Mass Index).

The following baseline parameters were collected for each patient before the start of the study: fasting blood glucose, glycated hemoglobin, creatinine, total cholesterol, LDL, Aspartate transaminase (AST), Alanine transaminase (ALT), height, weight, BMI calculation and blood pressure.

For a period of 15 days before the start of the study and for the entire 3-month duration of the study, each patient was required to record capillary blood glucose data at home in a special diary as follows: every second week on Monday, Wednesday and Friday, blood glucose measurement before and after lunch (2 h after the start of lunch). All study participants received the same device with all necessary materials for capillary blood glucose measurement. During the outpatient visit, each patient was given a diet with dietary instructions to maintain carbohydrate intake at lunch.

Throughout the study, participants were required to take 1 tablet, containing 500 mg of *Amaranthus caudatus* dry extract, at lunchtime.

### 2.9 Statistical analysis

Statistical analyses of *in vitro* assays and clinical data were performed using JMP Pro 14 (SAS Institute Inc.) and NCSS software. Data were summarized as mean ± standard deviation unless otherwise specified. Baseline comparisons were performed using Student’s *t*-test for paired data. For comparisons involving more than two groups, one-way analysis of variance (ANOVA) was applied, followed by appropriate *post hoc* tests when significant effects were detected. For all statistical tests, a *p* value < 0.05 was considered statistically significant. Graphical representations and summary tables were prepared using Microsoft Excel.

Exploratory multivariate analyses were performed using RStudio software (version 4.4.0). Correlation matrices were generated at baseline (T0) and after the intervention period (T1) using Spearman’s rank correlation coefficient. Principal Component Analysis (PCA) and unsupervised clustering analyses were applied as exploratory and visualization tools to investigate multivariate patterns and inter-individual variability. Clustering was performed on standardized data using k-means, with the number of clusters selected using a heuristic elbow method based on within-cluster variance. Multivariate analyses were conducted separately at T0 and T1 to allow qualitative comparison of patterns over time. Given the exploratory and observational nature of these analyses, results were not intended to support causal inference or confirmatory conclusions. The present study was designed as a preliminary experimental investigation combining *in vitro* assays and a pilot clinical before–after intervention without a parallel control group. Given the exploratory nature of the study and the absence of randomization and group comparison, no formal quality or risk-of-bias assessment using standardized tools was performed. Methodological rigor was ensured through standardized experimental procedures, predefined inclusion criteria, and consistent data collection at baseline (T0) and after the intervention period (T1).

## 3 Results

### 3.1 Characterization of Amaranthus extract through untargeted metabolomics

**Figure 2:**
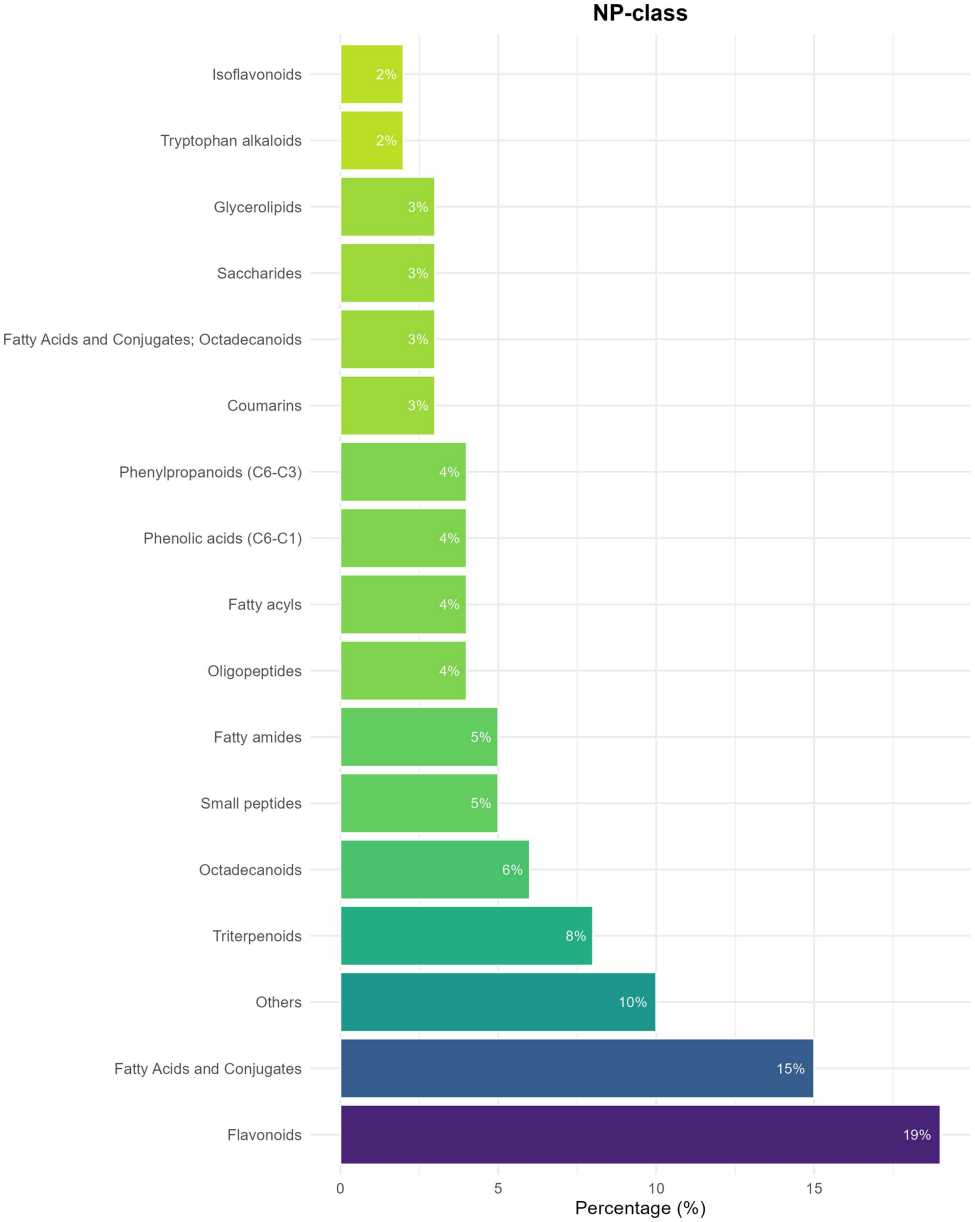
Natural product class. Distribution of annotated metabolites detected in the *Amaranthus caudatus* dry extract across major chemical classes. The histogram reports the percentage of compounds per chemical class, calculated relative to the total number of detected and annotated metabolites, and not based on peak areas or ion intensities. All metabolites shown correspond to putative identifications (MSI level 2) based on accurate mass and MS/MS spectral similarity.

Untargeted metabolomic analysis of the *Amaranthus caudatus* dry extract led to the identification of 174 metabolites (For the full list see “Supplementary material”), including 64 apolar (37%) and 111 polar compounds (63%), based on chromatographic behavior and ionization characteristics. All reported metabolites correspond to putative identifications (MSI Level 2), based on accurate mass and MS/MS spectral similarity to reference databases. The detected metabolites belong to several natural product classes. As shown in Figure 2, which reports the percentage distribution calculated on the total number of detected and annotated compounds per class, the most represented classes were flavonoids (19%), fatty acids and conjugates (15%), isoflavonoids (10%), and triterpenoids (8%), followed by smaller proportions of phenolic acids, fatty acyls, oligopeptides, and saccharides. This representation reflects compound counts only and does not imply quantitative abundance, as peak areas or ion intensities were not used for class comparison.

This compositional diversity highlights the complex chemical nature of the extract and its potential for multiple biological effects. Specifically, the flavonoids and phenolic compounds such as quercetin, naringenin and genistein detected in the dry extract likely account for its antioxidant properties, mainly through their ability to donate electrons or hydrogen atoms, thereby neutralizing reactive oxygen species (ROS) (21). In addition to direct radical scavenging, flavonoids can activate Nrf2-dependent pathways, upregulating endogenous antioxidant enzymes including superoxide dismutase (SOD), catalase (CAT), and glutathione (GSH) (22). Triterpenoids and conjugated fatty acids are closely associated with hypolipidemic and hypocholesterolemic effects. They can modulate intestinal lipid absorption by binding bile acids and cholesterol and regulate cholesterol metabolism through inhibition of HMG-CoA reductase or activation of AMPK, leading to decreased plasma LDL and triglyceride levels (23–25). Among triterpenoids, oleanane-type triterpenoid saponins were also identified. These compounds exhibit hypocholesterolemic activity, as they can bind bile salts and cholesterol in the intestinal lumen, thereby reducing their absorption. Extracts containing such saponins have been shown to lower plasma cholesterol levels in high-fat diet murine models (26). These associations are based on chemical class membership and literature evidence and are not intended to imply quantitative or causal relationships. Starting from this metabolomic characterization, a series of *in vitro* assays were conducted to evaluate the antioxidant, hypolipidemic, and hypocholesterolemic properties of the analyzed Amaranthus dry extract.

### 3.2 Assessment of the inhibition of *α*-amylase and α-glycosidase

**Figure 3:**
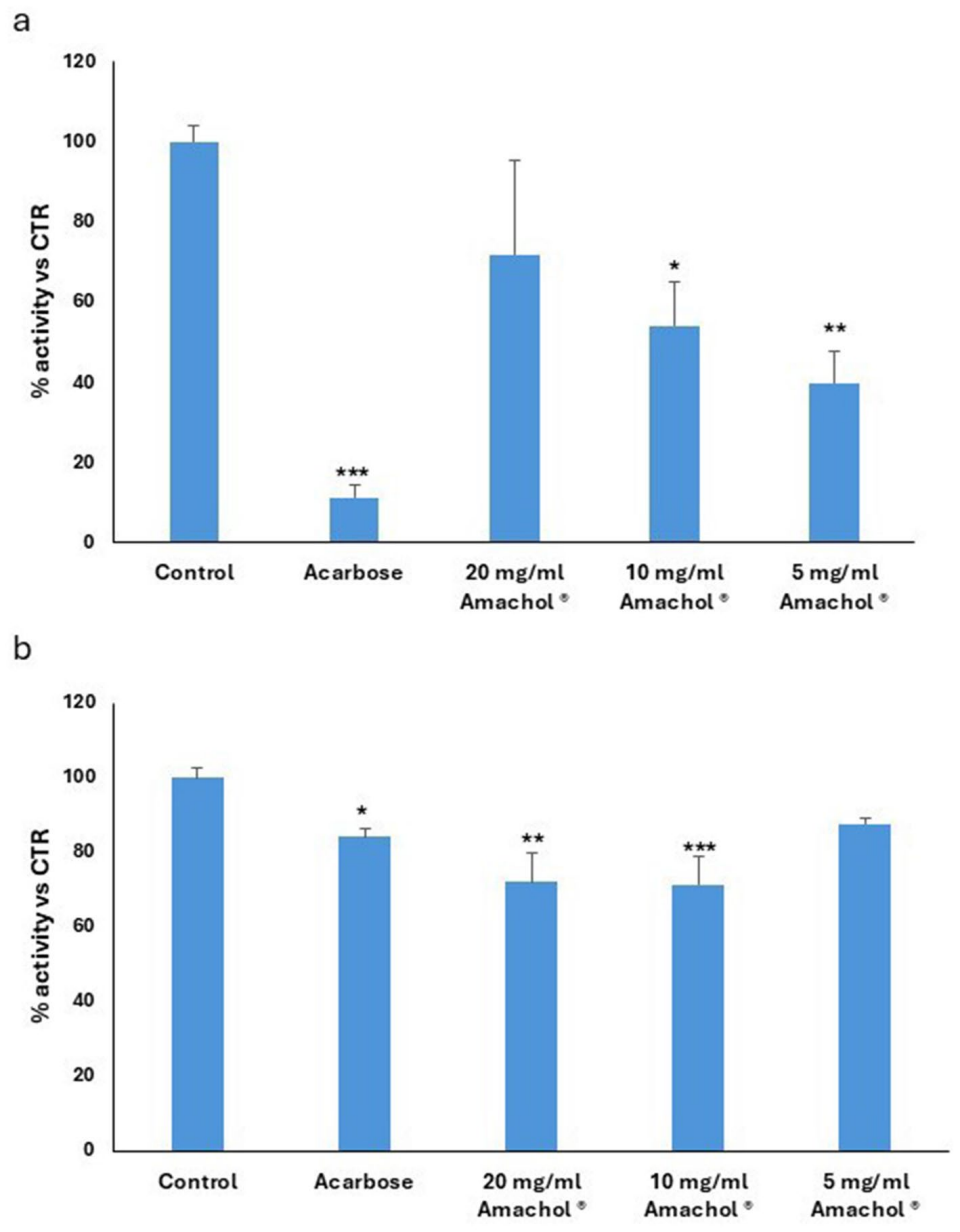
Evaluation of the inhibition of digestive enzymes involved in glucose release. **(a)** *α*-Amylase activity. Enzyme activity was measured in the absence of inhibitors (control) and in the presence of acarbose, used as a positive control. α-Amylase activity was assessed following incubation with three increasing concentrations of the *Amaranthus caudatus* dry extract. Results are expressed as percentage of residual enzymatic activity relative to the control group. Statistical significance was evaluated by one-way ANOVA, using the control group as the reference for multiple comparisons (*p* < 0.05, * *p* < 0.01, ** *p* < 0.001). **(b)** α-Glucosidase enzyme activity. Enzyme activity was measured in the absence of inhibitors (Control) and in the presence of acarbose, used as a positive control. α-Amylase activity was assessed following incubation with three increasing concentrations of the *Amaranthus caudatus* dry extract. Results are expressed as percentage of residual enzymatic activity relative to the control group. Statistical significance was evaluated by one-way ANOVA, using the control group as the reference for multiple comparisons (*p* < 0.05, * *p* < 0.01, ** *p* < 0.001).

The potential direct inhibiting activity toward the enzyme *α*-amylase and α-glycosidase were assessed by enzymatic assay. *Amaranthus caudatus* dry extract was tested at different concentrations (20–10 – 5 mg/mL) and α-amylase and α-glycosidase activity were measured. Acarbose was tested at a concentration of 1 mg/mL as positive control of enzyme inhibition. Results were expressed as % of activity versus untreated control. As shown in Figure 3a, *Amaranthus caudatus* extract exhibited a statistically significant inhibition on the *α*-amylase activity. Specifically, the results showed a reduction in enzyme functionality of 45 and 75% for the 10 and 5 mg/mL concentrations, respectively. On the contrary, the highest concentration did not show any statistically significant difference with untreated control. This non-linear behavior could suggest assay interference or substrate competition at high extract levels. These results indicate that *A. caudatus* dry extract can effectively inhibit *α*-amylase, potentially contributing to decreased postprandial glucose excursions. As shown in Figure 3b, *Amaranthus caudatus* dry extract exerted a statistically significant inhibitory action on α-glycosidase at the concentrations of 20 and 10 mg/mL, inducing a reduction of 30% concentrations tested. While less pronounced than α-amylase inhibition, this effect further supports the extract’s potential to modulate carbohydrate digestion and glucose release.

### 3.3 Antioxidant activity

**Figure 4:**
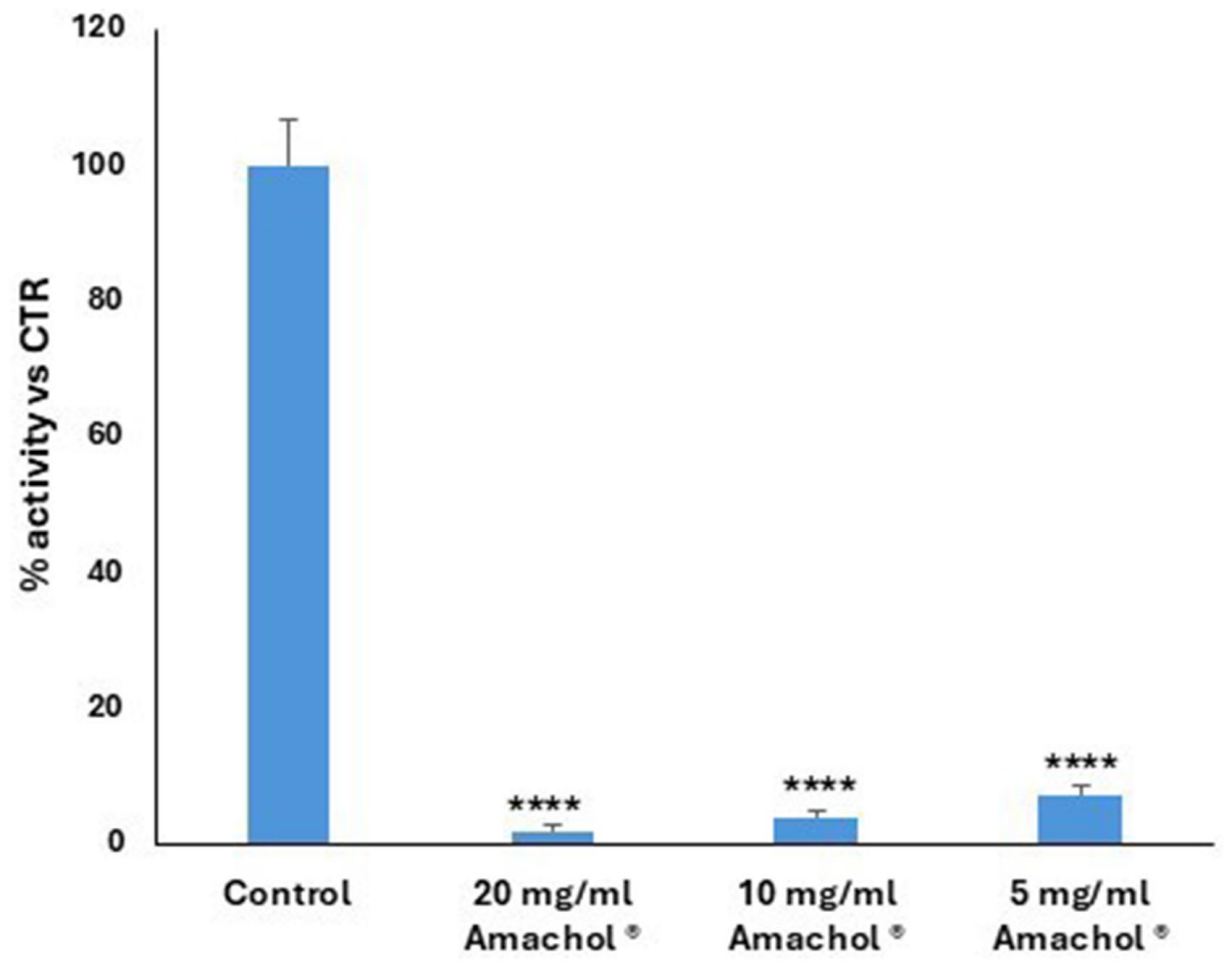
Antioxidant activity of AmaChol. Bar plot showing antioxidant activity measured in the presence of three different concentrations of AmaChol (20, 10, and 5 mg/mL). Values are expressed as percentage relative to the control. Data are presented as mean ± standard deviation. Statistical analysis was performed using one-way ANOVA, *** *p* < 0.0001.

The antioxidant potential of the extract was assessed by a colorimetric assay. Briefly, the capacity of preventing the oxidation of the reagent ABTS has been evaluated, and in Figure 4 the data showed that *Amaranthus caudatus* extract determined a very strong, dose-dependent antioxidant capacity. At every concentration tested (20–10 – 5 mg/mL) in a dose-dependent manner, the extract determined a significant reduction of ABTS oxidation, of 98, 96 and 93%, respectively. This potent activity aligns with the high flavonoid content identified in the metabolomic analysis and suggests that the extract can effectively neutralize ROS *in vitro*, providing a mechanistic basis for its potential protective effects against oxidative stress.

### 3.4 Analysis of free glucose release and intestinal absorption in an *in vitro* model

**Figure 5:**
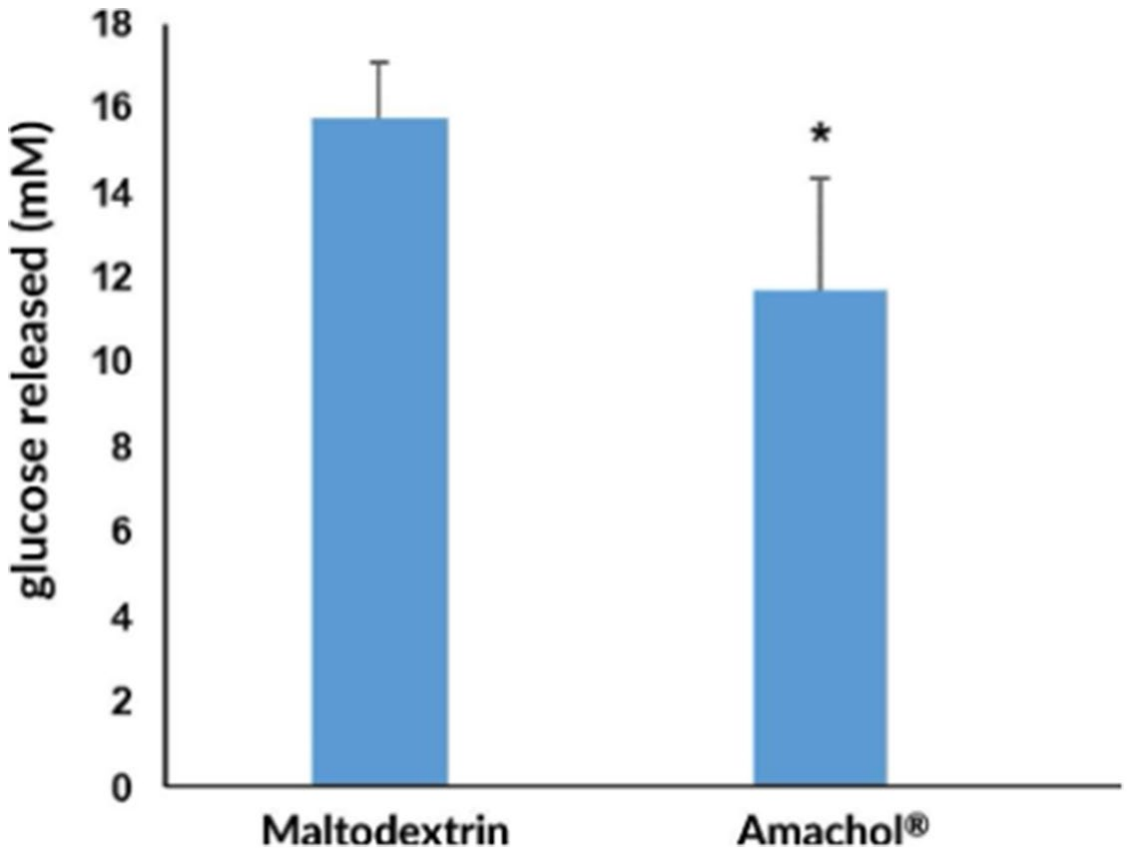
Free glucose release after simulated gastrointestinal digestion. *In vitro* gastrointestinal digestion was performed to evaluate free glucose release, which was quantified by a spectrophotometric assay. The graph shows glucose concentration expressed in mM and reported as mean ± standard deviation. Statistical analysis was conducted using a *t*-test, * *p* < 0.05.

The effect of *Amaranthus caudatus* dry extract on glucose availability was evaluated using an integrated *in vitro* model simulating human gastrointestinal digestion followed by intestinal absorption, according to Minekus et al. The impact of the extract was compared with maltodextrin, used as a positive reference. Specifically, maltodextrin was used because it is a well-characterized, rapidly digestible carbohydrate commonly employed as a carrier in nutraceutical formulations and exhibits a predictable glucose release profile during digestion. Its inclusion provides a standardized reference for interpreting the extent and kinetics of glucose liberation from AmaChol^®^ under identical in vitro digestive conditions. During simulated digestion, *A. caudatus* dry extract significantly reduced the amount of free glucose released from starch. As shown in Figure 5, glucose concentration reached 11.7 ± 2.65 mM, corresponding to a 26% reduction compared with maltodextrin (15.8 ± 1.28 mM; *p* < 0.05). This finding indicates a reduced extent of starch hydrolysis, consistent with the inhibitory effects observed on *α*-amylase and α-glucosidase activity.

**Figure 6:**
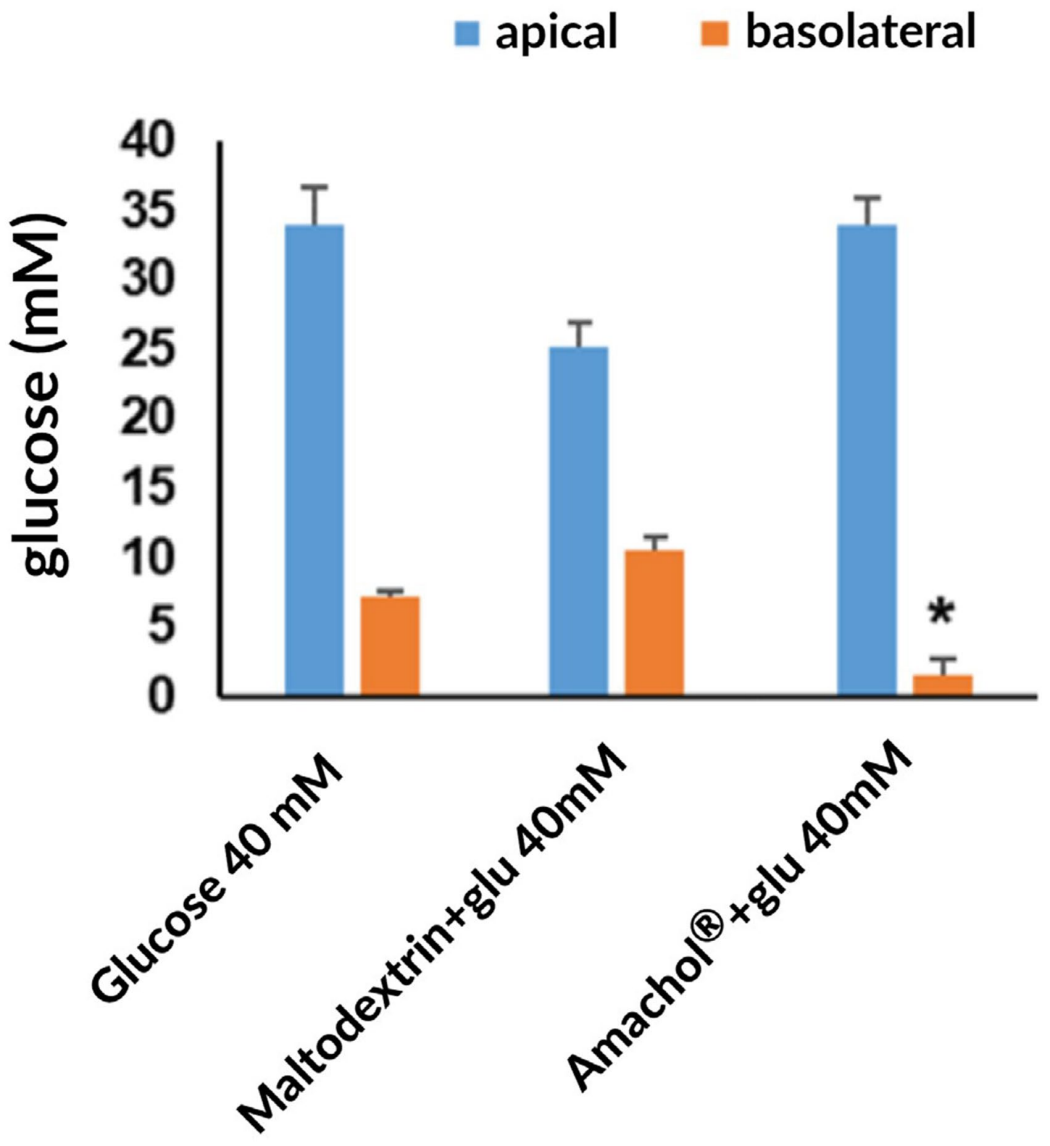
Evaluation of glucose absorption after simulated gastrointestinal digestion. Glucose absorption was assessed by quantifying glucose concentration in the basolateral compartment (absorbed fraction, orange bars) and in the apical compartment (unabsorbed fraction, blue bars) after 30 min of incubation. Data are reported as mean ± standard deviation. Statistical significance was assessed by one-way ANOVA, with maltodextrin + glucose used as the reference group for multiple comparisons, * *p* < 0.05.

Following digestion, intestinal absorption was assessed by incubating intestinal tissues with the digested matrices and quantifying glucose in the apical and basolateral compartments. The results are reported in Figure 6 and Table 1.

**Table 1:** Quantification of glucose absorption after gastrointestinal digestion.

Sample	Apical (mM)	Basolateral (mM)
Glucose 40 mM	33.9 ± 2.71	7.12 ± 0.46
Maltodextrin + glu 40 mM	24.2 ± 1.81	10.5 ± 1.05
AmaChol^®^ + glu 40 mM	34.0 ± 1.93	1.62 ± 1.10

Glucose concentration (mM) measured in the apical and basolateral compartments after 30 min incubation under different experimental conditions. Data are expressed as mean ± standard deviation.

Glucose absorption was significantly reduced in the presence of the *A. caudatus* dry extract compared with both maltodextrin and untreated control. In particular, basolateral glucose concentration after incubation with AmaChol^®^ was 1.62 ± 1.10 mM, compared with 10.5 ± 1.05 mM for maltodextrin, corresponding to an 85% reduction in glucose uptake.

Taken together, these results indicated that *A. caudatus* dry extract reduced glucose bioavailability by limiting both glucose release during digestion and subsequent intestinal transport, in agreement with the enzymatic inhibition data.

### 3.5 Insulin release assay from human pancreatic islets stimulated with simulated gastrointestinal digested and absorbed fractions of *Amaranthus caudatus* extract

**Figure 7:**
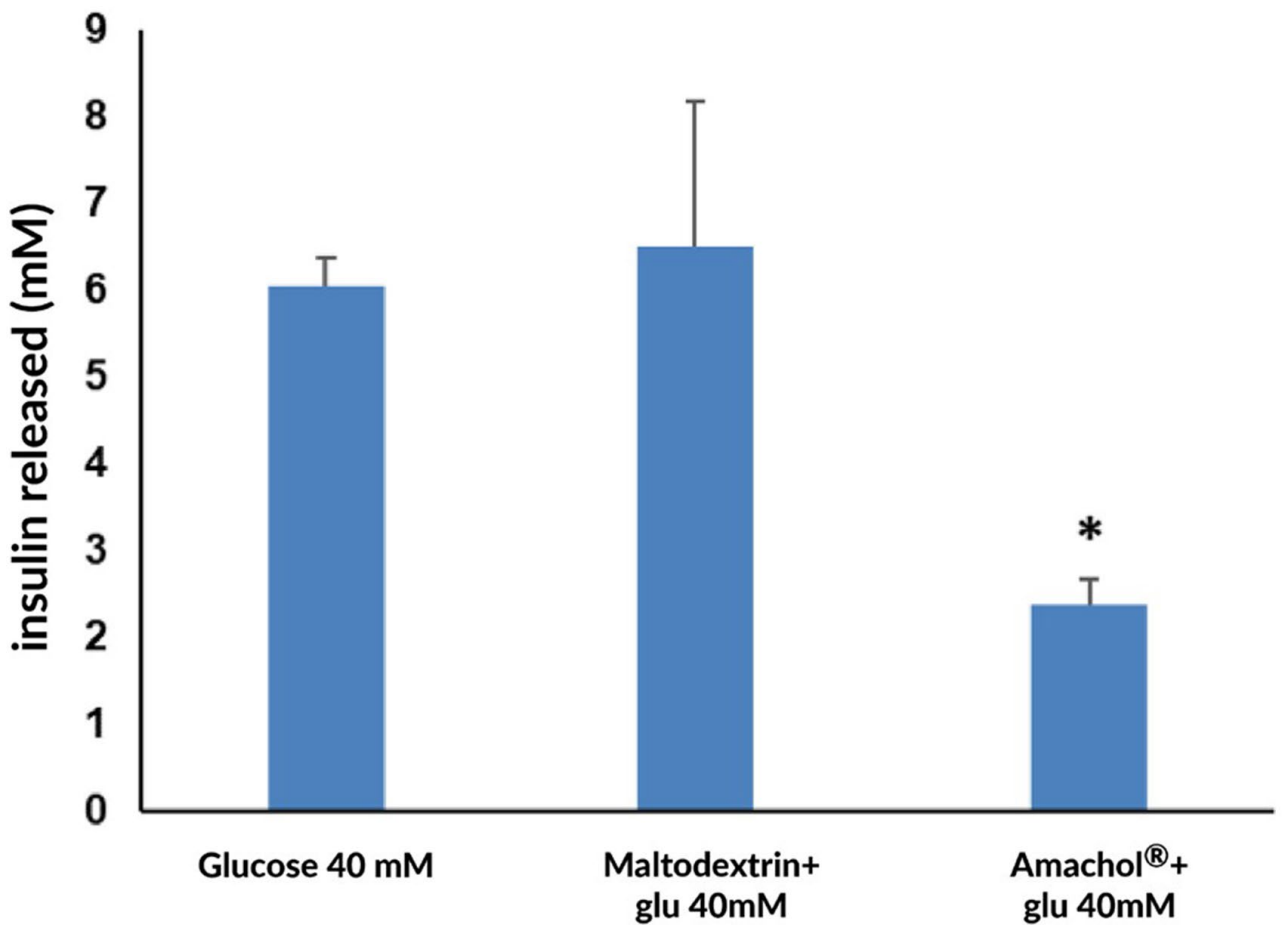
Quantification of insulin release from human pancreatic islets. Insulin secretion was measured in human pancreatic islets exposed to basolateral fractions obtained after *in vitro* gastrointestinal digestion and intestinal absorption. Insulin concentration is expressed in mM and reported as mean ± standard deviation. Statistical analysis was performed using one-way ANOVA, with maltodextrin + glucose used as the reference group for multiple comparisons, * *p* < 0.05.

After mimicking gastrointestinal digestion and intestinal absorption *in vitro*, the mechanisms inducing insulin release were simulated, in order to assess whether the treatment with *Amaranthus caudatus* dry extract would affect insulin release. Thus, pancreatic islets were exposed to basolateral fractions obtained from in vitro intestinal absorption, and insulin secretion was quantified by means of ELISA assay. As reported in Figure 7, *Amaranthus caudatus* dry extract induced a significant reduction of insulin release (2.38 ± 0.29 mM) if compared to maltodextrin (6.51 ± 1.67 mM) and to the control, corresponding to 63% reduction of insulin release. This reduction mirrored the lower glucose availability, suggesting that the extract’s inhibitory effect on carbohydrate digestion and absorption translates into decreased insulin demand.

### 3.6 Cholesterol absorption

**Figure 8:**
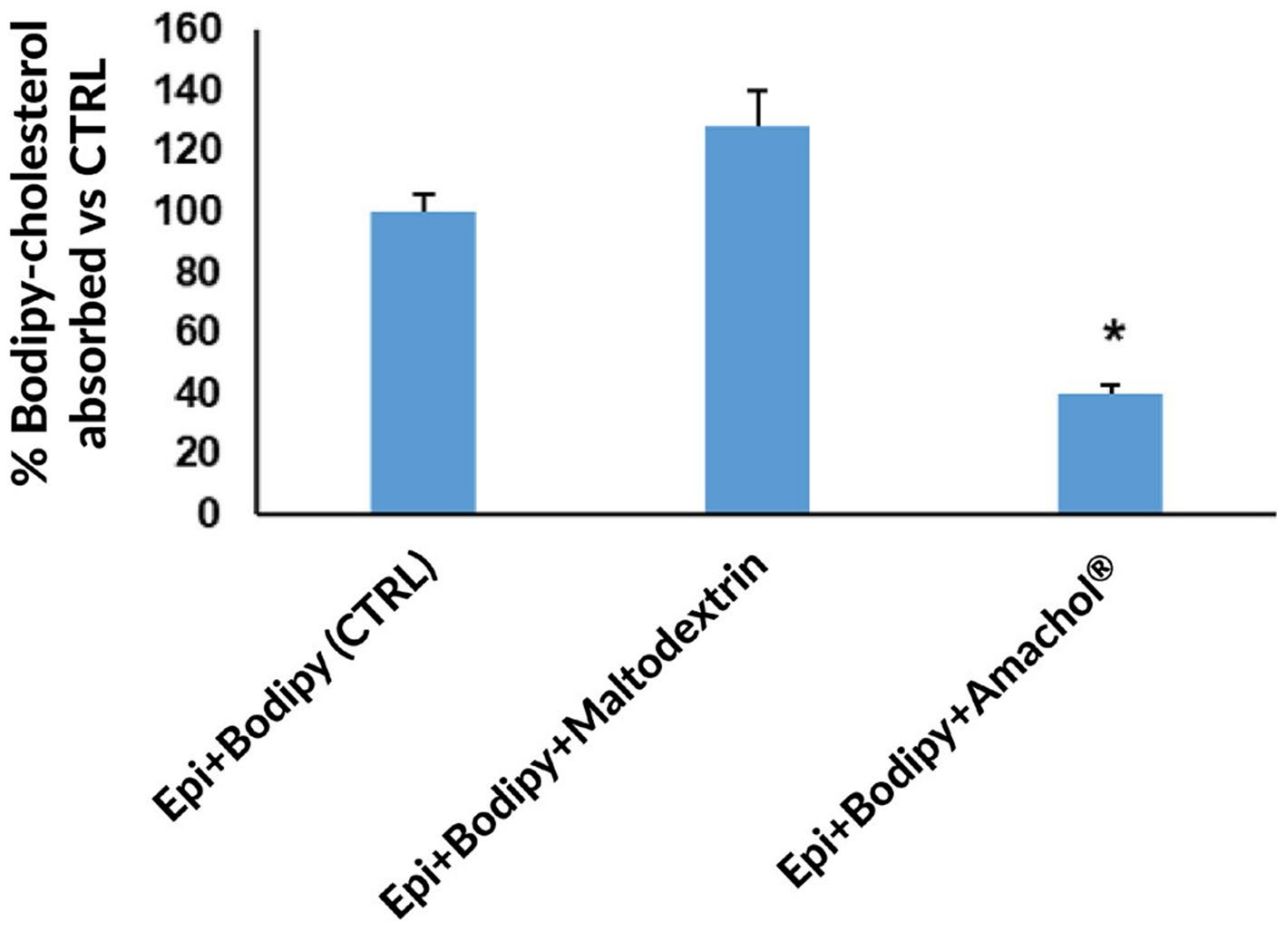
Quantification of cholesterol absorption. Cholesterol absorption was assessed by quantifying the fluorescent cholesterol analogue BODIPY-cholesterol in the basolateral compartment of the EpiIntestinal™ model. All bar plot values are expressed as percentage relative to the control (Epi + Bodipy). Data are reported as mean ± standard deviation. Statistical analysis was performed using one-way ANOVA, with the control group used as the reference group for multiple comparisons, * *p* < 0.05.

Moreover, the extract potential in lowering cholesterol intestinal absorption was assessed. The results obtained from the experiments demonstrated that dry extract significantly inhibited intestinal absorption of a fluorescent cholesterol analogue (BODIPY-cholesterol) by 69% when compared with maltodextrin and by 60% when compared with untreated control (BODIPY-only) (Figure 8). This finding is consistent with the presence of triterpenoids and conjugated fatty acids, which can interfere with cholesterol uptake and suggest potential hypolipidemic properties.

### 3.7 Preliminary observations in humans and statistical analysis

Preliminary observations in humans supporting the *in vitro* findings were conducted on a total of sixty patients with glucose intolerance and overweight (mean BMI 27.3 ± 3.2) followed over a period of 3 months to evaluate the effects of a dietary supplement based on amaranth on key metabolic syndrome parameters listed in Table 2.

**Table 2:** List of parameters measured in the preliminary observational study.

Abbreviation	Full parameter name
Age	Age
BMI T0	Body Mass Index at baseline (T0)
BMI T1	Body Mass Index after 3 months (T1)
PAmax T0	Systolic blood pressure at baseline (T0)
PAmax T1	Systolic blood pressure after 3 months (T1)
PAmin T0	Diastolic blood pressure at baseline (T0)
PAmin T1	Diastolic blood pressure after 3 months (T1)
Glc T0	Fasting blood glucose at baseline (T0)
Glc T1	Fasting blood glucose after 3 months (T1)
HbA1c T0	Glycated hemoglobin at baseline (T0)
HbA1c T1	Glycated hemoglobin after 3 months (T1)
COL T0	Total cholesterol at baseline (T0)
COL T1	Total cholesterol after 3 months (T1)
HDL T0	HDL cholesterol at baseline (T0)
HDL T1	HDL cholesterol after 3 months (T1)
LDL T0	LDL cholesterol at baseline (T0)
LDL T1	LDL cholesterol after 3 months (T1)
TGs T0	Triglycerides at baseline (T0)
TGs T1	Triglycerides after 3 months (T1)
AST T0	Aspartate transaminase (AST) at baseline (T0)
AST T1	Aspartate transaminase (AST) after 3 months (T1)
ALT T0	Alanine transaminase (ALT) at baseline (T0)
ALT T1	Alanine transaminase (ALT) after 3 months (T1)
GlcPRE	Pre-prandial blood glucose levels
GlcPOST	Post-prandial blood glucose levels

Abbreviated parameter names and their corresponding full descriptions.

At the end of the study, the number of patients for whom data were collected amounted to 37.

The mean age of the investigated sample was 54.2 ± 8.1 years (min = 31, max = 68 years) with median 53. Females (*n* 17, 45.9% of the total sample) were 57.6 ± 7.9 years old, whereas males (*n* 20, 54.1%) were 51.6 ± 7.4. Of the total cases, 75% are not older than 61 years and 90% are not older than 65. The modal age group is between 50 and 55 years with 44% of total cases. The study has been conducted as described in Section 2.5.

**Figure 9:**
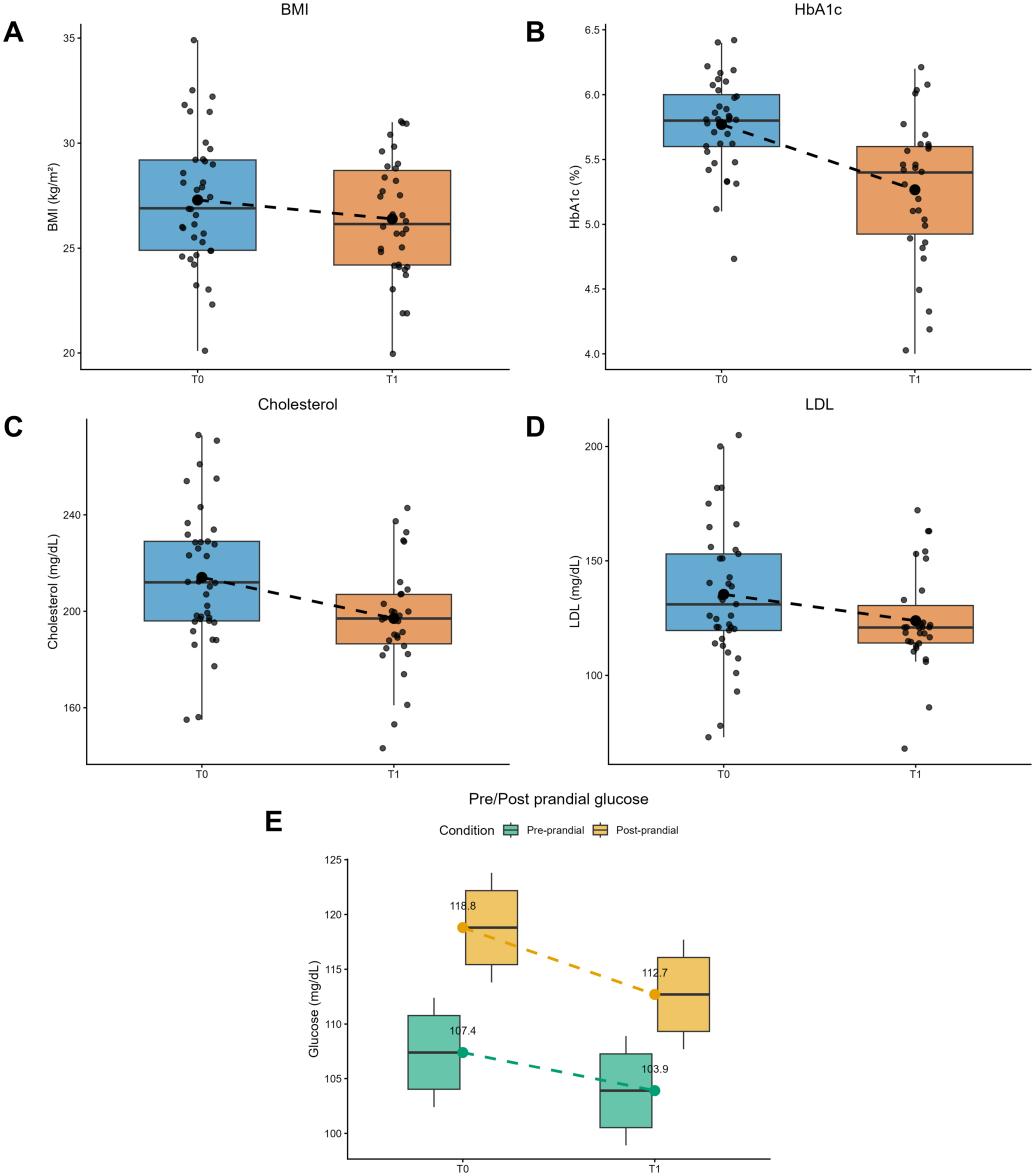
**(A)** Body mass index (BMI). Box plot of BMI values (kg/m^2^) among study participants at T0 (baseline) and T1 (after 3 months of treatment). Box plots represent the median, interquartile range, minimum and maximum values, individual data points, and standard deviation. **(B)** Glycated hemoglobin (HbA1c). Box plot of HbA1c values (%) at T0 and T1. Box plots represent the median, interquartile range, minimum and maximum values, individual data points, and standard deviation. **(C)** Total cholesterol. Box plot of total cholesterol values (mg/dl) at T0 and T1. Box plots represent the median, interquartile range, minimum and maximum values, individual data points, and standard deviation. **(D)** LDL cholesterol. Box plot of LDL values (mg/dl) at T0 and T1. Box plots represent the median, interquartile range, minimum and maximum values, individual data points, and standard deviation. **(E)** Blood glucose. Pre- and post-prandial blood glucose levels (green and yellow, respectively) at T0 and T1. Box plots represent the median, interquartile range, minimum and maximum values, and standard deviation.

Body mass index (BMI) showed a significant reduction after 3 months of treatment (T1), as shown in Figure 9A. Glycated hemoglobin (HbA1c), considered the primary end point, decreased significantly after 3 months of treatment (T1) from 5.8 ± 0.4 (T0) to 5.3 ± 0.6 (T1), as reported in Figure 9B. The secondary endpoints were pre- and post-prandial blood glucose, BMI, LDL, and total cholesterol. Regarding the lipid profile, the mean value of total cholesterol decreased from 212.16 mg/dL at T0 to 197.03 mg/dL at T1 (Figure 9C), while LDL cholesterol showed a reduction from 133.4 mg/dL at T0 to 123.86 mg/dL at T1 (Figure 9D); however, these changes were not statistically significant. Postprandial blood glucose levels significantly decreased after 3 months of supplement intake, whereas no significant difference was observed in basal (pre-prandial) blood glucose, despite a non-significant trend toward reduction (Figure 9E). Overall, when comparing the two time points, among the 28 patients with valid data, 50% showed an improvement in glycemic levels at the end of the study, while the remaining 50% were equally divided between patients who did not change their pathological status (25%) and those who worsened compared with baseline (25%).

**Figure 10:**
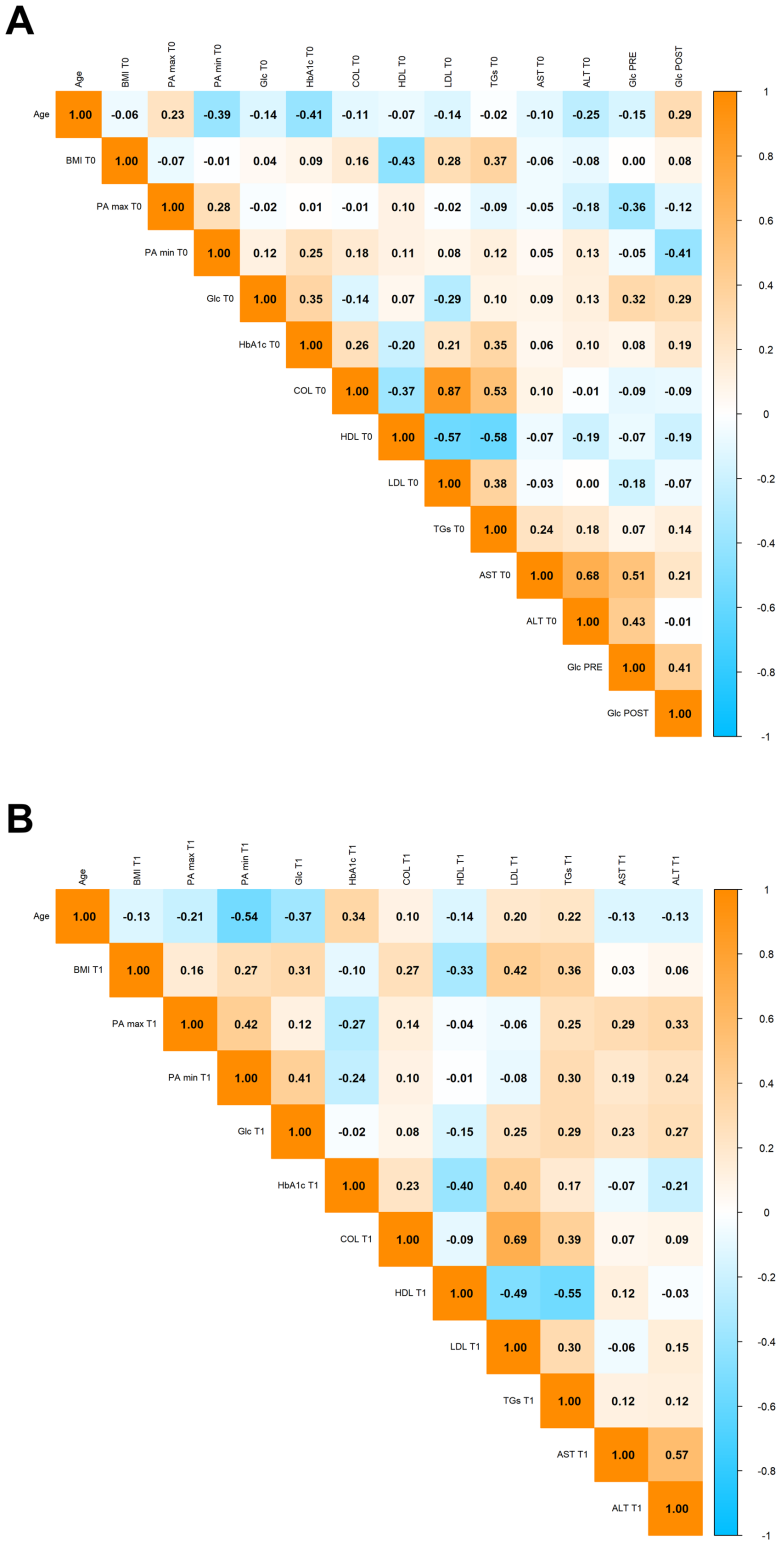
**(A)** Correlation matrix at baseline (T0). Heatmap representing pairwise correlations among variables at baseline. Correlation coefficients range from −1 to 1, with orange indicating the highest positive correlations and light blue indicating the lowest (or most negative) correlations. Box outlines and/or color intensity indicate the strength of each correlation. **(B)** Correlation matrix after the 3-month intervention period (T1). Heatmap representing pairwise correlations among variables after the 3-month intervention. Color scale is identical to panel **(A)** (orange = maximum positive correlation, light blue = minimum/negative correlation).

To investigate how the relationships among the parameters listed in Table 2 evolved over time, correlation matrices were generated at baseline (T0) (Figure 10A) and after the 3-month intervention period (T1) (Figure 10B).

At baseline, the correlation matrix revealed several expected associations that reflect the typical metabolic profile of patients with glucose intolerance. A strong positive correlation was observed between fasting blood glucose and glycated hemoglobin (HbA1c), which indicates that individuals with higher fasting glucose levels also tended to have higher long-term glycemic burden. Body weight and BMI were, as expected, closely correlated, and both showed positive associations with blood pressure values. This aligns with known links between excess weight and elevated cardiovascular risk. Furthermore, total cholesterol and LDL cholesterol also displayed a strong positive correlation, reflecting their shared role in lipid metabolism.

After 3 months of supplementation, the correlation matrix at T1 showed some interesting shifts. The positive association between fasting glucose and HbA1c remained present, although it appeared slightly attenuated, suggesting that some improvement in glycemic control may have occurred, possibly due to reduced postprandial glucose excursions. The correlation between weight and BMI remained stable, while the relationships between body weight and certain metabolic markers, such as HbA1c or lipid levels, seemed to become more pronounced. This could suggest that reductions in body weight were beginning to influence glycemic and lipid profiles more significantly.

Some inverse correlations also became more evident at T1. For instance, there appeared to be a stronger negative relationship between HbA1c and variables such as HDL cholesterol or body weight, indicating that patients who experienced improvements in glucose control also showed other signs of metabolic benefit. These evolving patterns hint at an overall rebalancing of metabolic parameters, likely supported by the effects of a dietary supplement based on amaranth extract.

In conclusion, while many of the baseline correlations remained consistent over time, the changes observed at T1 suggest that the intervention may have contributed to modulating some of the key metabolic relationships, particularly those involving glucose metabolism, lipid profile, and body weight. These findings support the potential role of the amaranth extract improving the metabolic profile of overweight individuals with glucose intolerance.

**Figure 11:**
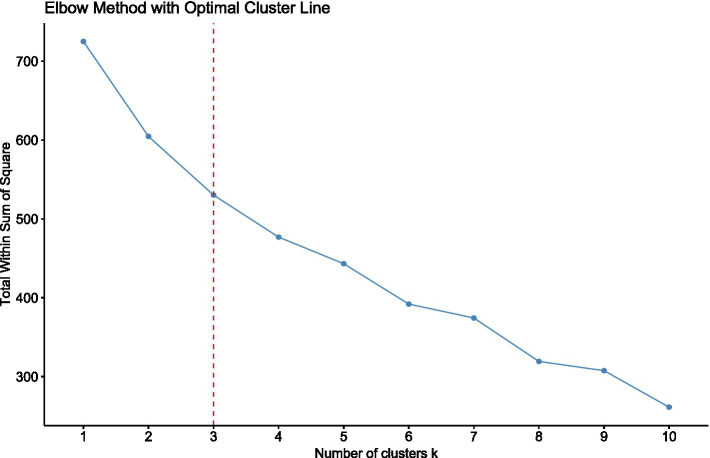
Elbow method for *k*-means clustering. The elbow method was applied to determine the optimal number of clusters. The *x*-axis reports the number of clusters (*k*), while the *y*-axis represents the total within-cluster sum of squares (WCSS). The vertical red dashed line indicates *k* = 3, corresponding to the elbow point where adding further clusters does not result in a substantial reduction of within-cluster variance.

To better understand the variability in patient response to the dietary intervention, a k-means clustering analysis was performed. This statistical technique allowed for the identification of subgroups of patients with similar baseline characteristics and patterns of metabolic change over time. By grouping individuals based on a combination of clinical and biochemical parameters (such as BMI, blood pressure, glucose levels, lipid profile, and liver enzymes) it was possible to explore whether certain profiles were associated with greater or lesser responses to treatment. Firstly, to determine the optimal number of clusters (K) for the k-means algorithm, the elbow method was applied. The optimal K turned out to be equal to 3 (Figure 11).

**Figure 12:**
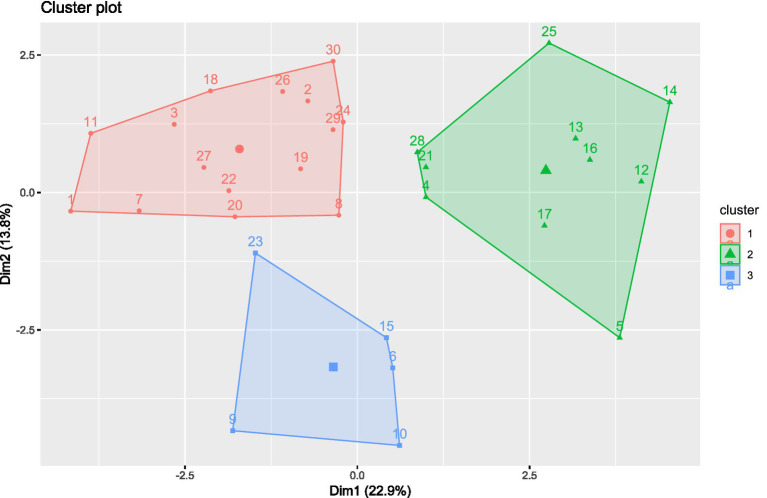
K-means clustering analysis. K-means clustering was performed using *k* = 3, as determined by the Elbow method. The plot shows the distribution of subjects along the first two dimensions (Dim1 and Dim2), explaining 22.9 and 13.8% of the total variance, respectively. Each point represents an individual subject, and colors indicate cluster membership (cluster 1: red; cluster 2: green; cluster 3: blue).

The resulting clusters offered valuable insights into which types of patients might benefit most from the amaranth extract used in the study (Figure 12).

Patients in *cluster 1* generally appear younger and tend to have a moderate BMI, typically below 29. Their baseline glycemic and lipid profiles are moderately altered, and over the course of the three-month study, they show clear improvements in several key parameters, including reductions in fasting blood glucose, HbA1c, and triglyceride levels. Liver enzyme values such as AST and ALT also tend to improve in many individuals within this group. Overall, cluster 1 seems to include patients with a relatively stable metabolic profile who nevertheless respond well to the intervention, suggesting good sensitivity to the supplement and dietary guidance.

In contrast, *cluster 2* includes patients who present more pronounced metabolic disturbances at baseline. These individuals often have higher BMI values, sometimes in the range of obesity class I, and show elevated levels of total cholesterol, LDL cholesterol, triglycerides, and HbA1c before treatment. However, what is particularly striking about this group is the magnitude of improvement observed over the study period. Patients in cluster 2 tend to experience the most significant reductions across multiple parameters, including marked decreases in HbA1c, postprandial glucose, and lipid values. This suggests that, despite starting from a higher-risk metabolic state, this group may benefit the most from the combined effect of the supplement and dietary recommendations.

*Cluster 3*, on the other hand, consists largely of older patients who tend to have a lower BMI compared to the other groups. Their baseline values are generally altered, with relatively controlled glucose and lipid levels at the start of the study. As a result, the changes observed over the 3 months are milder and less consistent. This subgroup appears to have a more stable, less reactive metabolic profile, and the intervention has a relatively limited impact. It is possible that these patients have less room for measurable improvement or that age-related factors influence the responsiveness to treatment.

In summary, cluster 1 includes younger individuals with moderate metabolic imbalance and good treatment response; cluster 2 comprises higher-risk patients who demonstrate the most substantial improvements; and cluster 3 includes older, leaner patients who show smaller, less consistent changes over time.

Overall, the chemical composition of *A. caudatus* extract, rich in flavonoids, triterpenoids, and phenolics, correlates with its observed biological activities: potent enzyme inhibition, antioxidant capacity, reduced glucose and cholesterol absorption, and modulation of insulin release. These findings provide a mechanistic link between extract composition and functional effects, highlighting the potential of *A. caudatus* as a modulator of metabolic processes in both vitro models and preliminary human observational studies. These observations provide useful insights into how different metabolic profiles respond to the same intervention and may be useful for future stratification of patients in larger and longer clinical trials.

## 4 Discussion and conclusion

*Amaranthus caudatus* is a pseudocereal that has attracted increasing interest in recent years, both for its nutritional profile (8) and for the numerous bioactivities that contribute to its therapeutic potential (9).

The untargeted metabolomic characterization of the specific *A. caudatus* seed extract (AmaChol^®^) revealed a chemically complex matrix composed of both polar and apolar metabolites, including flavonoids, isoflavonoids, triterpenoid saponins, fatty acids, phenolic acids, oligopeptides, and micronutrients. This compositional diversity is consistent with previous reports on amaranth seeds and supports the notion that the extract contains multiple classes of bioactive compounds.

The hypoglycaemic potential of the specific *A. caudatus* extract, as evidenced by *in vitro* studies, appears to involve multiple complementary mechanisms acting at the intestinal level. First, the observed inhibition of the carbohydrate-digesting enzymes *α*-amylase and α-glucosidase supports a direct luminal mechanism capable of delaying polysaccharide hydrolysis and glucose release. This finding is coherent with previous studies reporting inhibitory activity of amaranth-derived protein fractions and peptides against these enzymes and is biologically plausible given the high protein content of *A. caudatus* seeds (27–31).

Moreover, our experiments using an *in vitro* model of the human intestinal barrier demonstrated a reduction in glucose transport across the epithelial layer following exposure to the digested extract. This effect suggests a decreased intestinal permeability to glucose under postprandial-like conditions, which may contribute to attenuation of glucose flux into the systemic circulation. While the precise molecular targets involved cannot be identified within the present experimental framework, this observation provides an additional level of mechanistic support beyond enzyme inhibition alone.

In addition, exposure of human pancreatic islets to the *A. caudatus* extract resulted in a reduction in insulin secretion under the experimental conditions tested likely due to the combined action of phenolic compounds and soluble peptides. Since the limitations related to *in vitro* studies, a decrease in insulin release does not necessarily imply an improvement in insulin sensitivity, nor does it exclude the possibility of indirect or context-dependent effects. One plausible explanation is that reduced insulin secretion reflects a lower glucose stimulus, consistent with the intestinal effects described above, rather than a direct action on *β*-cell function. Supporting this, studies on other Amaranthus species have reported antidiabetic effects, including enhanced insulin sensitivity, which may be related to improved calcium homeostasis and *α*-amylase inhibition (32, 33).

**Figure 13:**
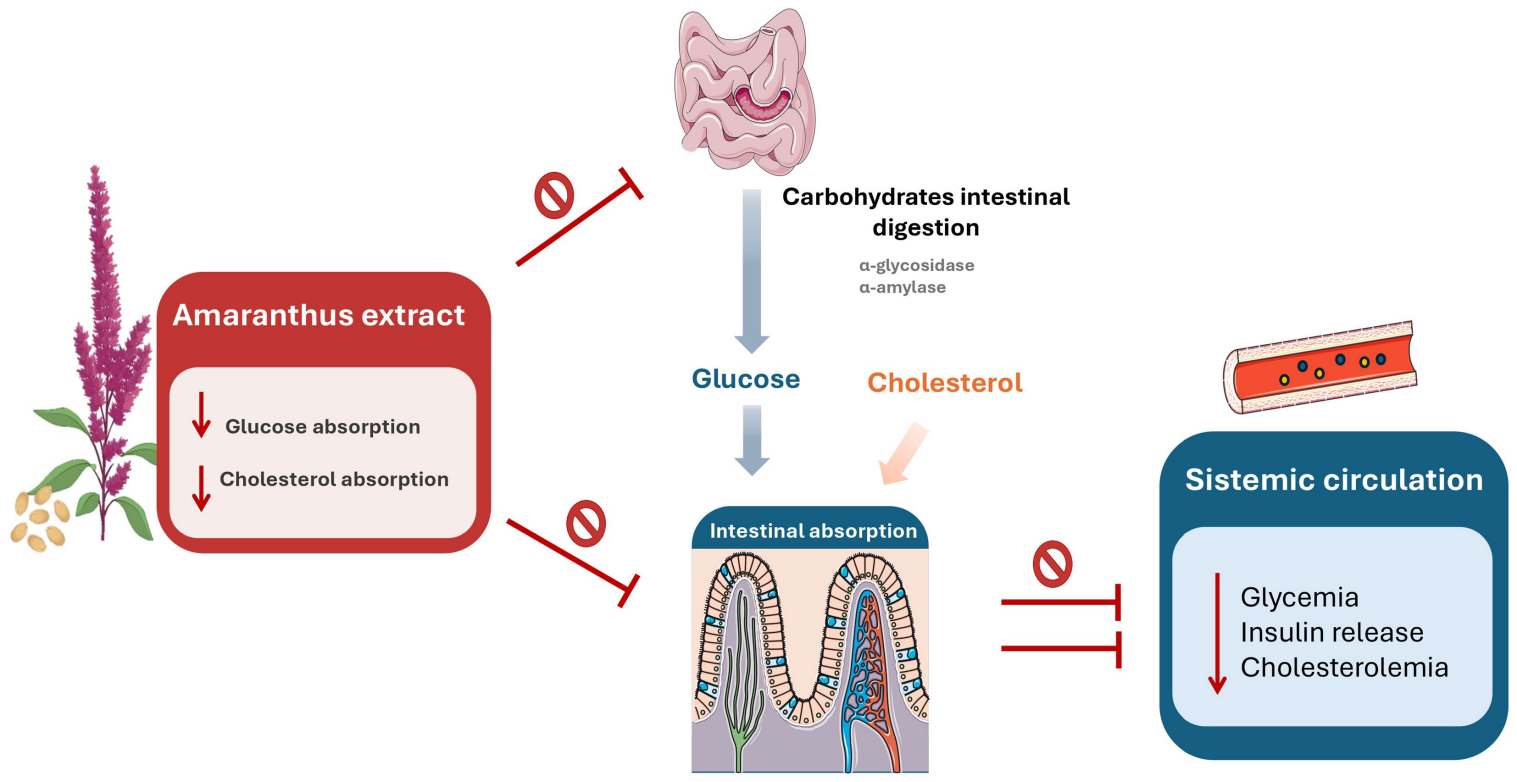
Proposed intestinal mechanisms contributing to the metabolic effects of *Amaranthus* extract. Schematic illustration of the possible pathways through which the *Amaranthus* extract may modulate glucose and lipid metabolism. The extract inhibits carbohydrate-digesting enzymes (α-amylase and α-glucosidase), leading to reduced glucose release and intestinal absorption. Independently, the extract inhibits intestinal cholesterol absorption without affecting digestive processes. The combined reduction in glucose and cholesterol uptake is associated with decreased glycemia, cholesterolemia, and insulin release. The figure represents a conceptual integration of the experimentally observed effects rather than a single, definitive mechanism of action. Image(s) provided by Servier Medical Art, licensed under CC BY 4.0 (43).

Additionally, the extract under investigation has been shown to inhibit intestinal cholesterol absorption in an in vitro model of human intestinal barrier. Previous studies have reported lipid-lowering effects of *A. caudatus*, particularly in terms of total cholesterol (TC) and low-density lipoprotein (LDL) cholesterol reduction (34–36). These beneficial properties can be attributed to the presence of soluble fibers, triterpenoid saponins, and protein-derived peptides, all of which have been implicated in lipid-lowering mechanisms (30). Indeed, previously published data and the data obtained from our metabolomic analysis clearly support the idea that extracts of amaranth may also contain components with hypolipidemic effects, since it has already been demonstrated that amaranth without its liposoluble fraction is still able to positively influence the lipid spectrum attributing this effect to the protein fraction (17, 22, 23) since some peptides seem to inhibit the activity of HMG-CoA reductase. Moreover, the metabolomic analysis revealed also the presence in this specific extract of naringenin that, from previous *in vivo* studies, resulted a metabolite involved in the lipid peroxidation and in the inhibition of HMG-CoA reductase, pathways involved in the reduction of cholesterol (37). In addition, oleanane-type triterpenoid saponins resulted from the characterization of AmaChol^®^ possess well-documented hypocholesterolaemic properties leading to a reducing absorption of the cholesterol that can be correlated with our *in vitro* studies (26). Moreover, the fiber contained in the extract can impaired the absorption of cholesterol from the digestive tract, for this reason, it is considered another possible component contributing to the hypolipidemic effect of amaranth extract on cholesterol (34–36). The combined inhibition of digestive enzymes, reduction of intestinal glucose transport, modulation of insulin secretion, and attenuation of cholesterol absorption suggests an integrated intestinal mechanism of action, schematically summarized in Figure 13. Further investigation will be performed in order to deeply explore the relationship between the class of compounds and the beneficial effect of the extract.

Beyond glycaemic and cholesterol control, the *A. caudatus* extract also exhibits significant antioxidant properties *in vitro* which may represent a modulatory mechanism influencing both glucose and lipid metabolism. This effect was related probably to the presence of specific polyphenols, flavonoids and micronutrients. For example, the metabolite calenduloside E exhibited potent antioxidant and anti-inflammatory activity in non-alcoholic fatty liver disease (NAFLD) models (38). Moreover, another metabolite resulted from untargeted analysis, the quercetin, showed strong radical scavenging activity and enhanced the activity of endogenous defense enzymes (22). Furthermore, the presence in this specific extract of genistein and pantothenic acid could contribute to the antioxidant activity since a reduction of ROS generation and an antioxidant capacity was attribute from the literature to both metabolites (39, 40, 41). For this reason, AmaChol^®^ could help to mitigate oxidative stress in metabolic dysfunction and cardiovascular risk.

In addition to the *in vitro* studies, a preliminary observational study was conducted, which confirmed and further expanded the findings on *A. caudatus* extract. Specifically, the observational study on amaranth extract supplementation provides preliminary yet valuable insights into its potential metabolic benefits in overweight individuals with glucose intolerance. Over a three-month period, significant improvements were observed in key marker such as glycated hemoglobin, postprandial glucose, and BMI, supporting the extract’s role in glycemic control and weight management. Although changes in lipid profiles were modest and not statistically significant, trends suggested a favorable shift. Different from *in vitro* study, the reduction in cholesterol after 3 months of treatment did not reach statistical significance. However, the average cholesterol level decreased below the clinical threshold with reduced dispersion toward higher values compared to baseline. This is an important trend as a starting point for further findings. Additional correlation analyses revealed evolving metabolic interrelationships, with some improvements linked to reductions in body weight. K-means clustering further highlighted differential responses among subgroups, with Cluster 2, comprising higher-risk individuals, showing the most pronounced benefits.

In conclusion, *A. caudatus* seed extract exhibits multiple biological activities *in vitro* that are mechanistically consistent with a potential role in modulating postprandial glucose handling and, to a lesser extent, lipid metabolism. Preliminary human observations support further investigation. The extract should therefore be considered a promising candidate for future preclinical and clinical studies in order to demonstrate its therapeutic efficacy.

### 4.1 Limitations and future steps

This study should be considered basically as a proof of concept on an extract of *Amaranthus caudatus*. The experiments, carried out in vitro and complemented by an observational data in subjects with altered glycemic control, suggest that *Amaranthus caudatus* seed extract may influence glucose and lipid metabolism through intestinal mechanisms and redox modulation. However, the evidence remains preliminary. The in vitro and observational studies do not allow attribution of biological effects to specific metabolites. Furthermore, the observational human study was exploratory with no placebo group, involving a modest number of subjects and was limited in the time. Moreover, no evaluation of cytotoxicity and safety was performed, therefore, the tolerability profile and margin of safety remain undefined. Future research must include standardized toxicological evaluations, randomized controlled trial designs with adequate sample sizes, predefined primary endpoints, and systematic dose–response analyses, ideally within longer-term studies. Nevertheless, given the growing demand for new nutraceutical products capable of supporting therapies for metabolic syndrome, and considering the promising preliminary results, this work provides a valuable starting point for future research in the field.

## Data Availability

The original contributions presented in the study are included in the article/Supplementary material, further inquiries can be directed to the corresponding author.
